# Impact of genetic profiles on periventricular anastomosis following bypass surgery in moyamoya disease

**DOI:** 10.1007/s10143-026-04289-8

**Published:** 2026-04-20

**Authors:** Seiei Torazawa, Satoru Miyawaki, Hideaki Imai, Hiroki Hongo, Masahiro Shimizu, Hideaki Ono, Shotaro Ogawa, Yu Sakai, Satoshi Kiyofuji, Satoshi Koizumi, Daisuke Komura, Hiroto Katoh, Shumpei Ishikawa, Nobuhito Saito

**Affiliations:** 1 Department of Neurosurgery, Faculty of Medicine, The University of Tokyo, 7-3-1 Hongo, Bunkyo-Ku, Tokyo, 113-8655 Japan; 2 Department of Neurosurgery, Tokyo Shinjuku Medical Center, Tokyo, Japan; 3 Department of Neurosurgery, Kanto Neurosurgical Hospital, Saitama, Japan; 4 Department of Neurosurgery, Fuji Brain Institute and Hospital, Shizuoka, Japan; 5 Department of Preventive Medicine, Graduate School of Medicine, The University of Tokyo, Tokyo, Japan

**Keywords:** Bypass surgery, Choroidal anastomosis, Moyamoya disease, Periventricular anastomosis, Rare Variant, *RNF213*

## Abstract

Postoperative regression of periventricular anastomosis (PA) may have important implications for preventing hemorrhagic events in patients with moyamoya disease (MMD). We aimed to identify the genetic factors associated with postoperative PA regression. This retrospective cohort study enrolled 81 patients (102 hemispheres) who underwent combined revascularization surgery. PA types (lenticulostriate, choroidal, and thalamic) were scored from 0 to 2 preoperatively and postoperatively using magnetic resonance angiography, and postoperative regression was evaluated. We extracted all exonic variants in *RNF213* and analyzed differences in their effects on PA regression between the p.Arg4810Lys variant and other *RNF213* variants. Among the 81 participants, 51 (63.0%) carried p.Arg4810Lys (all heterozygotes: GA group), whereas 20 (24.5%) harbored other rare or damaging *RNF213* variants (GG with other variants group). All types of PA regressed significantly in the GA group (*P* < 0.001 for all), whereas only choroidal PA showed significant regression in the GG with other variants group (*P* = 0.024). The postoperative decrease in choroidal PA score was greater in the GG with other variants group than in the GA group (*P* = 0.058). Multivariate analysis using linear mixed-effect models suggested a potential association between choroidal PA score reduction and two factors: the GG genotype with other variants and age < 16 years. Our findings indicate that harboring *RNF213* variants other than p.Arg4810Lys may be associated with greater regression of choroidal PA compared with harboring p.Arg4810Lys in this exploratory cohort. Comprehensive genetic analysis of *RNF213* may enable more accurate prediction of the surgical preventive effect on hemorrhagic events in MMD.

**Supplementary Information:**

The online version contains supplementary material available at 10.1007/s10143-026-04289-8.

## Introduction

Moyamoya disease (MMD) is a rare cerebrovascular disorder characterized by progressive stenosis at the terminal portion of the internal carotid artery and marked development of collateral vessels [1, 2]. Periventricular anastomosis (PA), defined as well-developed moyamoya vessels connecting with medullary or insular arteries in the periventricular area, is another significant feature of MMD and is classified into three groups based on the perforating arteries from which they arise: lenticulostriate, choroidal, and thalamic [3, 4]. In particular, choroidal PA is recognized as an important risk factor for intracranial hemorrhagic events in MMD [5, 6].

PA regression after revascularization surgery in MMD has been reported in multiple studies [7–9]. In particular, emphasis has been placed on the regression of choroidal PA after revascularization surgery, as it may indicate effective mitigation of intracranial hemorrhage risk through surgery [5, 8, 10]. Identifying predictive factors for significant PA regression supports the development of optimal treatment strategies in MMD. However, key patient-specific factors associated with postoperative PA regression remain poorly understood.

Regarding genetic analyses, the association between genetic variants and postoperative PA regression has been investigated in only one report to date [11] and therefore remains elusive. That study analyzed only one variant: *RNF213* c.14429G > A (p.Arg4810Lys), which is a well-established risk variant for MMD [12, 13]. In addition to p.Arg4810Lys, associations between other rare variants in *RNF213* and MMD have also been reported [14–16]. Although the functional consequences of both p.Arg4810Lys and other *RNF213* rare variants on RNF213 protein activity remain unclear, accumulating evidence suggests that different *RNF213* variants may influence diverse clinical phenotypes of MMD. A research group recently reported that there may be significant differences in the clinical effect between p.Arg4810Lys and other *RNF213* variants. Torazawa et al. found that p.Arg4810Lys wild-type cases with *RNF213* rare variants had a higher risk of hemorrhagic events than p.Arg4810Lys-positive cases [17]. Furthermore, they recently reported significant differences in postoperative donor artery development among different *RNF213* genotypes [18]. Given these findings, we hypothesized that *RNF213* variants other than p.Arg4810Lys may influence surgical effects, including PA regression, in a genotype-dependent manner.

In this study, we aimed to elucidate the significant preoperative factors that can predict favorable postoperative PA regression. We performed a comprehensive variant analysis of *RNF213* using exome sequencing and analyzed the difference in their effect on PA regression, particularly between the patients carrying p.Arg4810Lys and those with other *RNF213* variants.

## Materials and methods

### Participants

This retrospective cohort study included patients with MMD who consented to genetic analysis and revascularization surgery at our institution between October 2011 and August 2023. MMD was diagnosed according to the latest criteria of the Japanese Research Committee on Moyamoya Disease [19]. Combined bypass using direct superficial temporal artery, middle cerebral artery anastomosis with encephalo-myo-synangiosis was performed in all cases, following a previously reported method [20], with minor modifications. This study was reported in accordance with the STROBE guidelines (Online Resources). Clinical trial number: not applicable. This study was approved by the institutional research board (approval number: G10026; approval date: September 12, 2011). This study was performed in accordance with the ethical standards as laid down in the 1964 Declaration of Helsinki and its later amendments or comparable ethical standards.

### Clinical and imaging data collection

The clinical data collected are presented in Table 1, and the diagnostic criteria and definition of each data point are described in Online Resource 1. In this study, a cutoff age of < 16 years was adopted in accordance with the definition of pediatric patients by the Japanese Society of Pediatric Neurosurgery.

**Table 1:** Preoperative characteristics of all 102 hemispheres

	All hemispheres (*n* = 102)	Group 1: *RNF213* GA (*n* = 65, 63.7%)	Group 2: *RNF213* GG with other variants (*n* = 13, 12.7%)	Group 3: without any *RNF213* variant (*n* = 24, 23.5%)
Sex (female)	72 (70.6)	49 (75.4)	9 (69.2)	14 (58.3)
Age at operation				
Median [IQR] (years)	44 [34.3–52]	43 [35–52]	44 [33–53]	47 [37–50.3]
< 16 years	10 (9.8)	10 (15.4)	0 (0.0)	0 (0.0)
Hypertension	32 (31.4)	20 (30.8)	6 (46.2)	6 (25.0)
Diabetes mellitus	5 (4.9)	3 (4.6)	2 (15.4)	0 (0.0)
Dyslipidemia	25 (21.6)	11 (16.9)	5 (38.5)	6 (25.0)
Smoking	26 (25.5)	12 (18.5)	3 (23.1)	11 (45.8)
Preoperative Gr2 PA				
Lenticulostriate	31 (30.4)	20 (30.8)	4 (30.8)	7 (29.2)
Choroidal	52 (51.0)	35 (53.8)	5 (38.5)	12 (50.0)
Thalamic	22 (21.6)	19 (29.2)	1 (7.7)	2 (8.3)
PCA involvement	25 (24.5)	19 (29.2)	3 (23.1)	3 (12.5)
Suzuki stage				
2	7 (6.9)	6 (9.2)	0 (0.0)	1 (4.2)
3	76 (74.5)	45 (69.2)	10 (76.9)	21 (87.5)
4	18 (17.6)	14 (21.5)	3 (23.1)	1 (4.2)
5	1 (1.0)	0 (0.0)	0 (0.0)	1 (4.2)
Preoperative symptom				
TIA	59 (57.8)	42 (64.6)	6 (46.2)	11 (45.8)
Infarction	29 (28.4)	16 (24.6)	5 (38.5)	8 (33.3)
Hemorrhage	14 (13.7)	7 (10.8)	2 (15.4)	5 (20.8)

Data are presented as *n* (%) or median [IQR]

In the hemisphere-based analysis, no hemisphere showed two or more preoperative symptoms simultaneously

*GA* Heterozygote of p.Arg4810Lys; *GG* Wild-type of p.Arg4810Lys; *IQR* Interquartile range; *PCA* Posterior cerebral artery; *TIA* Transient ischemic attack

### Evaluation of PA and postoperative bypass development

For PA evaluation, we used magnetic resonance angiography (MRA) images reformatted as a sliding thin-slab maximum intensity projection and diagnosed each PA type (lenticulostriate, choroidal, and thalamic) according to the criteria reported by Funaki et al. [4]. Grading of each PA type (range, 0–2) was based on the definition by Funaki et al. [21]. In general, a grade 2 PA is regarded as a positive indicator of each PA type, reflecting a significant connection to the medullary or insular artery [4, 21]. In this study, we defined the grade of each PA as the “PA score” and calculated the total PA score (range, 0–6) by summing the individual PA scores (range, 0–2) for the three PA types in each hemisphere.

**Fig. 1:**
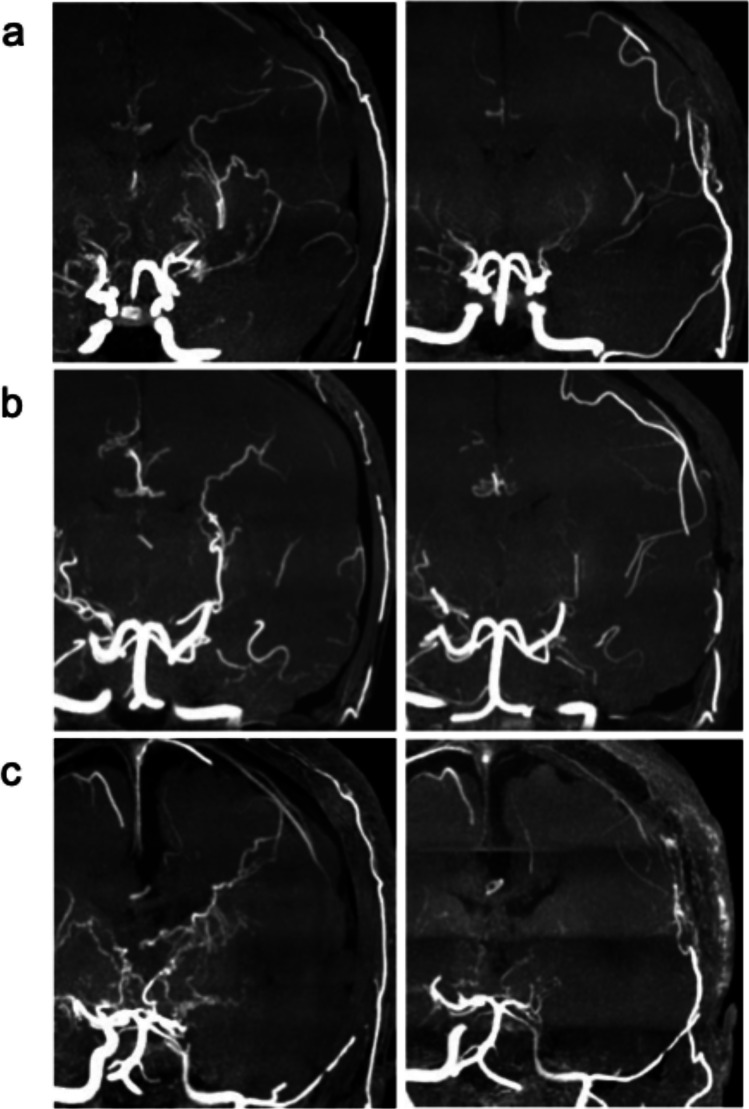
Preoperative and postoperative magnetic resonance angiography images of each type of periventricular anastomosis (PA). Representative examples of favorable PA regression are shown in all three types of PA: **a** lenticulostriate, **b** choroidal, and **c** thalamic. (left) Preoperative images depicting grade 2 PA. (right) Postoperative images depicting grade 0 PA

For postoperative bypass development evaluation, we assessed the superficial temporal artery, middle meningeal artery, and deep temporal artery (DTA) by comparing preoperative and postoperative MRA images (the grading system used in this study is described in the Online Resources). The grading of each PA and postoperative bypass development was assessed by two experienced neurosurgeons (S.T. and S.O.), who were blinded to the patient’s genotypes and clinical information during the image evaluation. Representative MRA images of postoperative regression of each PA type are presented in Fig. 1. Postoperative MRA evaluations of PA and bypass development were performed between 6 months and 1 year after surgery. When multiple postoperative MRAs were available for a given patient, the examination closest to 1 year after surgery was selected for analysis to ensure consistency in evaluation timing.

### Sequencing of all exonic variants in *RNF213*

We used *RNF213* sequencing data extracted from whole-exome sequencing generated using the Twist Comprehensive Exome Panel Kit (Twist Bioscience, South San Francisco, CA, USA) and NovaSeq6000 (Illumina, San Diego, CA, USA). The variant calling process, performed based on the human reference genome (Genome Reference Consortium Human Build 38 [GRCh38]), and extraction of the *RNF213* region were conducted as previously reported [17]. We defined and extracted “rare” and/or “damaging” variants in *RNF213* (Online Resource 1).

### Statistical analyses

All statistical analyses were performed using SPSS version 26 (IBM Corp., Armonk, NY, USA) and R studio version 2026.01.0 + 392 (Posit Software, Boston, MA, USA). Weighted kappa was calculated to assess the interrater agreement on the evaluation of PA grading and postoperative bypass development. Changes in PA scores before and after surgery were evaluated using the Wilcoxon signed-rank test. To account for within-patient clustering of bilateral hemispheres, mixed-effects models were applied with patient identity included as a random effect. Linear mixed-effects models were used for continuous variables, and the appropriate generalized mixed-effects models were used for binary and ordinal outcomes. Multiple comparisons were adjusted using Dunnett’s method. Statistical significance was set at *P* < 0.05.

## Results

In this study, we enrolled 81 participants (102 operated hemispheres). The median age at initial surgery was 44 years (interquartile range, 37–52 years), and 57 (70.4%) participants were female. Regarding the results of genetic analysis, 51 (63.0%) were p.Arg4810Lys heterozygous (GA), 30 (37.0%) were wild-type (GG), and none were homozygous. Aside from p.Arg4810Lys, *RNF213* rare variants or damaging variants were detected in 20 patients (24.5%) (Online Resource 4).

We divided all enrolled hemispheres into the following three groups: group 1, GA; group 2, GG with other variants; group 3, no *RNF213* variants (Table 1). Among patients with GA, nine patients also harbored other *RNF213* variants. Because *RNF213* p.Arg4810Lys is a well-established hotspot variant that is strongly associated with MMD, these patients were classified into group 1, assuming a predominant effect of the p.Arg4810Lys variant. The variants detected in group 2 were p.Gly2440Asp, p.Asp2554Glu, p.Arg2704Gln, p.His3204Arg, p.Met3666Thr, p.Pro4250Thr, p.Ala4399Thr, and p.Glu4950Asp (Online Resource 5). The basic characteristics of all enrolled hemispheres and the differences among the three groups are summarized in Table 1.

Preoperative grade 2 lenticulostriate PA was observed in 31 hemispheres (30.4%), choroidal PA in 52 (51.0%), and thalamic PA in 22 (24.5%). Preoperative hemispheric symptoms included transient ischemic attacks in 59 hemispheres (57.8%), cerebral infarctions in 29 (28.4%), and hemorrhages in 14 (13.7%). The mean duration from surgery to postoperative MRA evaluation was 230 days (range, 180–360 days). No patients experienced postoperative intracranial hemorrhagic events.

### Reliability of image evaluation

The weighted kappa values for the grading of PA and postoperative bypass development were 0.67 and 0.61, respectively. In cases of disagreement between raters, the final evaluations were determined through consensus discussion conducted in a blinded manner, without access to the patient’s genotype or other clinical information.

### Postoperative regression of PA in all hemispheres

We analyzed postoperative regression of PA using the Wilcoxon signed-rank test across all enrolled hemispheres. The mean decrease in total PA score was 1.55 ± 1.29, with decreases of 0.70 ± 0.68 in lenticulostriate PA, 0.80 ± 0.79 in choroidal PA, and 0.91 ± 0.72 in thalamic PA. All reductions were statistically significant (*P* < 0.001 for all comparisons; Online Resource 3), indicating that combined revascularization surgery is associated with significant regression in all types of PA.

### Analysis of postoperative change for each genotype group

We then analyzed postoperative bypass development and PA regression according to the *RNF213* genotype group. As the primary objective of this study was to examine differences between groups 1 (GA) and 2 (GG with other *RNF213* variants), we used mixed-effects models and Dunnett’s method for multiple comparisons, designating group 1 as the reference group (Table 2). The postoperative development score for DTA was significantly lower in group 2 than in group 1 (*P* = 0.030). Conversely, a greater reduction in choroidal PA score was observed in group 2 (1.5 ± 0.55) compared with group 1 (0.74 ± 0.74); however, this difference did not reach statistical significance (*P* = 0.058). No statistically significant differences were observed when comparing group 3 (no *RNF213* variants) to group 1.

**Table 2:** Postoperative bypass development and PA regression in each *RNF213* genotype group (hemisphere-level analysis using mixed-effects models)

	Group 1: *RNF213* GA (*n* = 65)	Group 2: *RNF213* GG with other variants (*n* = 13)	*P* value (vs. GA)	Group 3: without any *RNF213* variant (*n* = 24)	*P* value (vs. GA)
Bypass development score					
STA	1.62 ± 0.60	1.38 ± 0.65	0.50	1.42 ± 0.72	0.31
MMA	0.88 ± 0.76	1.00 ± 0.82	0.89	0.83 ± 0.82	0.97
DTA	1.38 ± 0.78	0.77 ± 0.93	**0.030**	1.12 ± 0.90	0.26
Total grading			0.61		** < 0.001**
Fair	4 (6.2)	2 (15.4)		2 (8.3)	
Good	34 (52.3)	10 (76.9)		16 (66.7)	
Excellent	27 (41.5)	1 (7.7)		6 (25.0)	
PA regression					
PA score decrease					
Lenticulostriate	0.70 ± 0.67	0.88 ± 0.99	0.71	0.62 ± 0.51	0.88
Choroidal	0.74 ± 0.74	1.5 ± 0.55	0.058	0.69 ± 0.95	0.98
Thalamic	1.00 ± 0.71	0.50 ± 0.71	0.57	0.67 ± 0.82	0.70
Total PA score	1.59 ± 1.29	1.89 ± 1.54	0.72	1.24 ± 1.15	0.51

Data are presented as mean ± standard deviation or n (%)

*P* values were calculated by Dunnett-adjusted comparisons. Values in bold indicate *P* < 0.05

*GA* Heterozygote of p.Arg4810Lys; *GG* Wild-type of p.Arg4810Lys; *STA* Superficial temporal artery; *MMA* Middle meningeal artery; *DTA* Deep temporal artery; *PA* Periventricular anastomosis

### Comparison between the genotypes of GA and GG with other variants

**Fig. 2:**
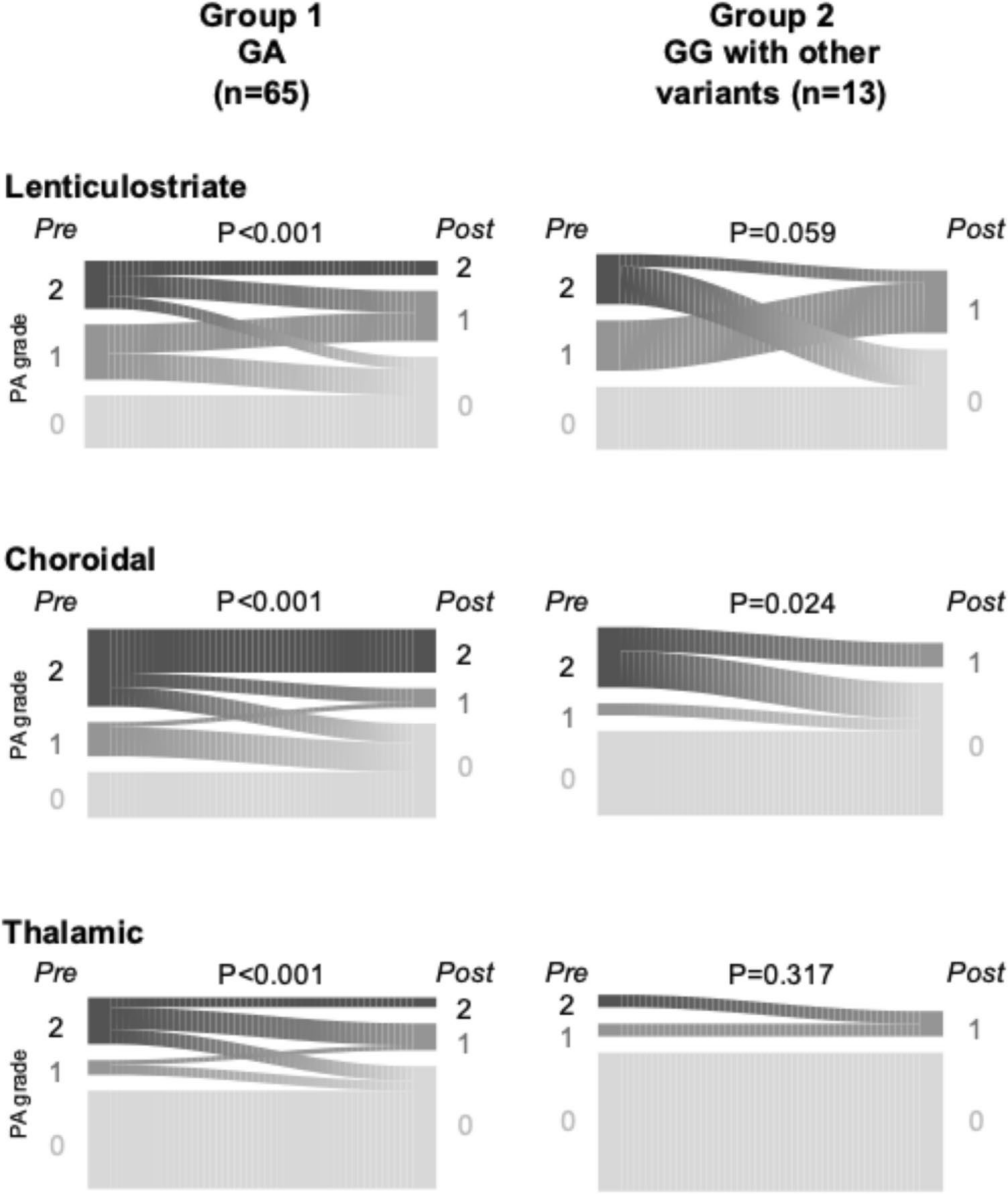
Postoperative change of all types of PA in groups 1 and 2. In group 1, all types of PA showed significant regression, whereas in group 2, only choroidal PA showed significant regression. GA, heterozygote of p.Arg4810Lys; GG, wild-type of p.Arg4810Lys; PA, periventricular anastomosis

**Fig. 3:**
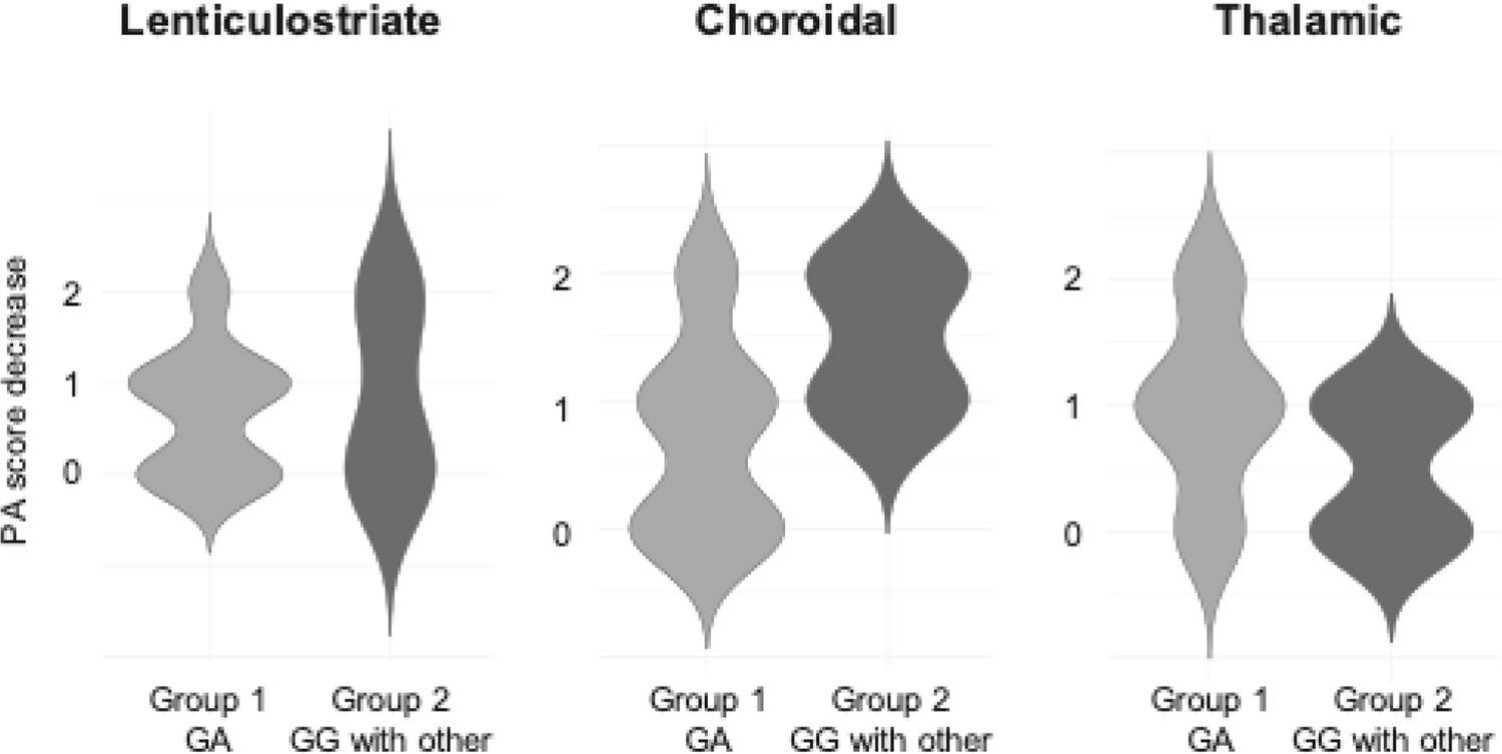
Violin plot depicting the postoperative score decrease of the three types of PA in groups 1 and 2. The score decrease of choroidal PA was greater in group 2 than in group 1 (*P* = 0.058). GA, heterozygote of p.Arg4810Lys; GG, wild-type of p.Arg4810Lys; PA, periventricular anastomosis

Subsequently, we compared groups 1 and 2 to further elucidate the differential effects of p.Arg4810Lys and other *RNF213* variants on PA regression (Fig. 2). The Wilcoxon signed-rank test revealed significant regression of all types of PA in group 1 (*P* < 0.001, for all), whereas only choroidal PA showed significant regression in group 2 (*P* = 0.024). As previously shown in Table 2, the decrease in choroidal PA score was greater in group 2 than in group 1 (Fig. 3).

Among patients in groups 1 and 2, univariate analyses using mixed-effect models were conducted to identify factors associated with the decrease in choroidal PA score. The analysis revealed that age at surgery < 16 years was the only significant factor (*P* = 0.003) (Online Resource 6). Other factors such as Suzuki grade or medical histories did not show a significant association with PA regression. Based on these findings, we performed multivariate analyses using liner mixed-effects models to identify factors associated with the decrease in choroidal PA score, including age at surgery (< 16 years), genotype group (group 1 vs. group 2), and postoperative DTA development (Table 3). The results showed that age < 16 years was associated with a smaller decrease in choroidal PA score (*P* = 0.012; coefficient, − 0.70; 95% confidence interval [CI], − 1.23 to − 0.17), and group 2 was associated with a greater decrease (*P* = 0.038; coefficient 0.62; 95% CI, 0.048 to 1.19).

**Table 3:** Multivariate analysis of factors associated with a decrease in choroidal PA score in the combined cohorts of groups 1 and 2 (linear mixed-effects model)

	*P* value	Coefficient B	95% CI
Age at surgery < 16 years (vs. > 16 years)	**0.012**	− 0.70	− 1.23 to − 0.17
Group 2: GG with other (vs. group 1: GA*)*	**0.038**	0.62	0.048 to 1.19
DTA development score	0.62	− 0.065	− 0.32 to 0.19

Values in bold indicate *P* < 0.05

*PA* Periventricular anastomosis; *CI* Confidence interval; *DTA* Deep temporal artery; *GG* Wild-type of p.Arg4810Lys; *GA* Heterozygote of p.Arg4810Lys

## Discussion

In this study, we conducted a comprehensive analysis of the *RNF213* variants using whole-exome sequencing and examined the association between *RNF213* genotypes and postoperative PA regression. Our findings demonstrated a significant difference in the surgical impact on choroidal PA regression between the GA group and the GG group harboring non-p.Arg4810Lys *RNF213* variants. Multivariate analysis suggested that both age at surgery and genotype grouping are potential factors associated with postoperative choroidal PA regression. Notably, these associations remained significant even after adjusting for postoperative bypass development, a factor previously reported to influence PA regression [11, 22–24]. Given that choroidal PA is a recognized risk factor for hemorrhage in MMD, our findings suggest that it may be possible to predict the preventive effect of surgical intervention against hemorrhagic events in specific genetically defined subgroups of MMD patients. These insights may hold potential for advancing personalized genotype-informed treatment strategies in MMD.

The regression of PA following revascularization surgery has been reported in several studies [7–9]. However, only a few significant factors associated with favorable postoperative regression of PA have been identified to date. One of these factors is the wider extent of the revascularized area after surgery. For instance, Zheng et al. reported this association in a pediatric cohort undergoing indirect bypass surgery [11], and Yamamoto et al. observed similar findings in a mixed cohort of children and adults treated with combined direct and indirect bypass [22]. Notably, Funaki et al. uniquely reported that PAs specifically targeted for regression significantly shrank postoperatively following the application of the tailored targeting bypass strategy [23]. In this approach, a direct bypass is deliberately placed to a cortical artery receiving blood flow from the PA via connected medullary arteries [23]. They subsequently demonstrated, through multivariate analysis, that effective revascularization of the region posterior to the central sulcus, where choroidal PAs predominantly drain, was significantly associated with favorable regression of these lesions [24]. Collectively, these findings suggest that PA can be normalized by reducing hemodynamic stress through successful and appropriately distributed revascularization. In contrast, we demonstrated that patients with GG carrying other *RNF213* variants showed significantly greater regression of choroidal PA despite poorer postoperative DTA development compared with the GA group. Although this appears contradictory to the conventional hemodynamic explanation, it suggests that genetic background might influence postoperative PA normalization through mechanisms that are not solely dependent on the extent of bypass development. Regarding the association between PA regression and genetic variants, only one study has been published to date. Zheng et al. analyzed a single *RNF213* variant, p.Arg4810Lys, in a cohort of pediatric patients who underwent indirect bypass surgery alone and found no significant association between the variant and PA regression [11]. Our current study is the first to explore the association between additional *RNF213* variants and postoperative PA regression.

It is important to note that the precise pathophysiological mechanisms by which *RNF213* variants influence intracranial artery phenotypes, as well as the reasons for the differing effects between p.Arg4810Lys and other *RNF213* variants, remain poorly understood. However, as discussed in previous reports [17, 18], growing evidence supports the clinical significance of *RNF213* variants beyond p.Arg4810Lys [14–18]. Integrating the current findings with prior observations, it is becoming increasingly clear that the potential impact of these less common variants cannot be overlooked. Further accumulation of clinical data on their implications is therefore essential. Ultimately, such efforts may lead to more accurate prediction of MMD clinical presentation through comprehensive genetic analysis of *RNF213*. In this context, the present study should be regarded as hypothesis-generating, highlighting a potential link between the *RNF213* genotype and postoperative vascular remodeling, rather than providing a definitive mechanistic explanation.

To elucidate the pathophysiological impact of *RNF213* variants in MMD development, it is important to note that most reported variants are located in the C-terminal region of the RNF213 protein. This observation suggests that these variants may affect the RING-finger domain, potentially leading to dysfunction of the RNF213 protein as an E3 ubiquitin ligase. *RNF213* functions as an E3 ubiquitin ligase and has been reported to target NFAT1, a transcription factor involved in the WNT signaling pathway and angiogenesis, as well as filamin A, a cytoskeletal protein implicated in vascular stenosis [25]. Given these molecular functions, it is plausible that *RNF213* variants are closely involved in the unique vascular phenotypes observed in MMD, including postoperative vascular remodeling after revascularization surgery. It is crucial to clarify the mechanism by which RNF213 dysfunction contributes to MMD pathogenesis through future in vitro studies, for example, by transfecting human umbilical vein endothelial cells with these *RNF213* variants and analyzing the resulting alterations in biological pathways. Future experimental approaches incorporating *RNF213* variant-specific models will be essential to bridge the gap between genetic findings and the present ones.

Regarding the association between age and postoperative PA regression, very few studies have explored this issue, and no clear consensus has been established to date. In the study by Funaki et al., a nonsignificant trend suggested that patients with substantial PA regression tended to be younger than those without [24]. In contrast, Zheng et al. reported that younger patients were more likely to exhibit postoperative progression of PA in a pediatric-only cohort [11]. These findings appear contradictory, and neither study reached statistical significance. In clinical practice, pediatric patients often exhibit imaging features suggesting a greater dependence of intracranial blood flow on moyamoya vessels rather than on major intracranial arteries. Under such conditions, even after revascularization surgery, PA may be less likely to regress. Therefore, the relationship between age and postoperative PA regression remains incompletely understood and likely reflects complex interactions between disease stage, vascular adaptability, and hemodynamic dependency.

This exploratory study has several limitations. First, this was a retrospective cohort study, and we enrolled only patients in whom whole-exome sequencing could be performed. As a result, patient enrollment was nonconsecutive, introducing a potential selection bias. Second, the sample size was limited, particularly in the non-p.Arg4810Lys variant group, and validation of our findings in larger, independent, and more diverse cohorts is essential. Furthermore, baseline characteristics differed between the study groups, and some patients exhibited an overlap of variants (e.g., harboring both p.Arg4810Lys and other rare variants). Although multivariate analyses were performed to adjust for these potential confounders, residual confounding and limited statistical power cannot be fully excluded, and these factors preclude the establishment of a definitive biological mechanism or direct causality. Third, although postoperative MRA evaluations were restricted to a follow-up window between 6 and 12 months after surgery, variability in the exact timing of imaging remained, which may have influenced the observed degree of PA regression. Fourth, postoperative clinical symptoms such as the frequency of transient ischemic attacks, as well as postoperative hemodynamic changes assessed by cerebral perfusion imaging, were not evaluated. Additionally, short- and long-term clinical outcomes were not assessed; therefore, we cannot determine whether the factors associated with PA regression are predictive of symptomatic improvement, hemodynamic recovery, or a favorable long-term neurological prognosis. Finally, although statistically significant differences in choroidal PA regression were observed among genotype groups, the magnitude of change was relatively modest, and its clinical relevance remains uncertain. Therefore, the present findings are exploratory and hypothesis-generating, requiring confirmation in larger independent cohorts. They should not be directly interpreted as evidence of clinical benefit; instead, future studies should focus on integrating genetic, radiological, hemodynamic, and clinical outcome data to clarify the clinical significance of PA regression.

In the GG group harboring *RNF213* variants other than p.Arg4810Lys, only choroidal PA exhibited significant postoperative regression. Moreover, these non-p.Arg4810Lys *RNF213* variants were identified as a factor associated with greater choroidal PA regression compared with p.Arg4810Lys. These findings suggest that genetic background could potentially serve as a factor in predicting the surgical impact on PA regression. However, as an exploratory study, further large-scale research is required to determine the clinical utility of comprehensive *RNF213* analysis for guiding individualized treatment strategies in patients with MMD.

## Supplementary Information

Below is the link to the electronic supplementary material.

Supplementary file1 (DOCX 23 KB)

Supplementary file2 (DOCX 438 KB)

Supplementary file3 (DOCX 247 KB)

Supplementary file4 (DOCX 17 KB)

Supplementary file5 (DOCX 19 KB)

Supplementary file6 (DOCX 17 KB)

## Data Availability

The data supporting the findings of this study are available from the corresponding author upon reasonable request from any investigator.
