# Supplementary material for: Impact of genetic profiles on periventricular anastomosis following bypass surgery in moyamoya disease
Source: Neurosurg Rev. 2026 Apr 20;49(1):363. doi: 10.1007/s10143-026-04289-8 (PMC13092527; doi:10.1007/s10143-026-04289-8)
Supplement: Supplementary file 1 — Supplementary file1 (DOCX 23 KB) [file 10143_2026_4289_MOESM1_ESM.docx]

**Impact of Genetic Profiles on Periventricular Anastomosis Following Bypass Surgery in Moyamoya Disease**

Seiei Torazawa^1^, Satoru Miyawaki^1^, Hideaki Imai^1,2^, Hiroki Hongo^1^, Masahiro Shimizu^3^, Hideaki Ono^1^, Shotaro Ogawa^1^, Yu Sakai^1^, Satoshi Kiyofuji^1,4^, Satoshi Koizumi^1^, Daisuke Komura^5^, Hiroto Katoh^5^, Shumpei Ishikawa^5^, Nobuhito Saito^1^

^1^The University of Tokyo, Department of Neurosurgery, Faculty of Medicine, Tokyo, Japan

^2^Tokyo Shinjuku Medical Center, Department of Neurosurgery, Tokyo, Japan

^3^Kanto Neurosurgical Hospital, Department of Neurosurgery, Saitama, Japan

^4^Fuji Brain Institute and Hospital, Department of Neurosurgery, Shizuoka, Japan

^5^The University of Tokyo, Department of Preventive Medicine, Graduate School of Medicine, Tokyo, Japan

**Corresponding author:** **Satoru Miyawaki, MD, PhD**

E-mail: smiya-nsu@m.u-tokyo.ac.jp

**Online Resource 1**

Diagnostic criteria for each underlying disease:

- Hypertension: systolic blood pressure ≥140 mmHg or the use of antihypertensive drugs.
- Diabetes mellitus: fasting blood glucose >126 mg/dL, casual blood glucose >200 mg/dL, or the use of hypoglycemic drugs.
- Dyslipidemia: low-density lipoprotein cholesterol in fasting blood ≥140 mg/dL, high-density lipoprotein cholesterol <40 mg/dL, triglyceride level >150 mg/dL, or oral treatment with lipid-lowering drugs.

Definition of each clinical data:

- Hemorrhagic symptoms included intracerebral hemorrhage, intraventricular hemorrhage, and subarachnoid hemorrhage.
- Posterior cerebral artery involvement was defined as >50% occlusion or stenosis in segments P1–P3, based on time-of-flight magnetic resonance angiography images.

Definition of “rare” or “damaging” variants in *RNF213*:

- For each extracted variant, allele frequency was analyzed using the Genome Aggregation Database based on the entries in dbSNP (<https://www.ncbi.nlm.nih.gov/snp/>), and variant annotation was performed using ANNOVAR (<https://annovar.openbioinformatics.org/en/latest/>, updated on 2023-03-15).
- A variant was defined as “rare” when its minor allele frequency was <0.01 in the Genome Aggregation Database.
- A variant was defined as “damaging” only if it met both of the following criteria simultaneously: its combined annotation-dependent depletion score (GRCh38-v1.6) was >10, and it was categorized as “deleterious/damaging or probably deleterious/damaging” by one or more of the following four prediction tools: Sorting intolerance from Tolerant, PolyPhen-2, MutationTaster, and Protein Variation Effect Analyzer.

Grading system for postoperative bypass development evaluation:

- Grade 0, postoperative signal intensity weakened or unchanged; grade 1, slight postoperative increase in signal intensity; grade 2, marked postoperative increase in signal intensity (**Online Resource 2**).
- Then, we defined the “total grade” as follows: fair, grade 2 in neither direct bypass nor indirect bypass (middle meningeal artery or deep temporal artery); good, grade 2 in either direct or indirect bypass; excellent, grade 2 in both direct and indirect bypass.
- For the postoperative evaluations, we primarily used imaging obtained between 6 months and 1 year after surgery.
