# Supplementary material for: Impact of genetic profiles on periventricular anastomosis following bypass surgery in moyamoya disease
Source: Neurosurg Rev. 2026 Apr 20;49(1):363. doi: 10.1007/s10143-026-04289-8 (PMC13092527; doi:10.1007/s10143-026-04289-8)
Supplement: Supplementary file 3 — Supplementary file3 (DOCX 247 KB) [file 10143_2026_4289_MOESM3_ESM.docx]

**Impact of Genetic Profiles on Periventricular Anastomosis Following Bypass Surgery in Moyamoya Disease**

Seiei Torazawa^1^, Satoru Miyawaki^1^, Hideaki Imai^1,2^, Hiroki Hongo^1^, Masahiro Shimizu^3^, Hideaki Ono^1^, Shotaro Ogawa^1^, Yu Sakai^1^, Satoshi Kiyofuji^1,4^, Satoshi Koizumi^1^, Daisuke Komura^5^, Hiroto Katoh^5^, Shumpei Ishikawa^5^, Nobuhito Saito^1^

^1^The University of Tokyo, Department of Neurosurgery, Faculty of Medicine, Tokyo, Japan

^2^Tokyo Shinjuku Medical Center, Department of Neurosurgery, Tokyo, Japan

^3^Kanto Neurosurgical Hospital, Department of Neurosurgery, Saitama, Japan

^4^Fuji Brain Institute and Hospital, Department of Neurosurgery, Shizuoka, Japan

^5^The University of Tokyo, Department of Preventive Medicine, Graduate School of Medicine, Tokyo, Japan

**Corresponding author:** **Satoru Miyawaki, MD, PhD**

E-mail: smiya-nsu@m.u-tokyo.ac.jp

**Online Resource 3** PA regression among all enrolled hemispheres. PA, periventricular anastomosis
