# Supplementary material for: Impact of genetic profiles on periventricular anastomosis following bypass surgery in moyamoya disease
Source: Neurosurg Rev. 2026 Apr 20;49(1):363. doi: 10.1007/s10143-026-04289-8 (PMC13092527; doi:10.1007/s10143-026-04289-8)
Supplement: Supplementary file 4 — Supplementary file4 (DOCX 17 KB) [file 10143_2026_4289_MOESM4_ESM.docx]

**Impact of Genetic Profiles on Periventricular Anastomosis Following Bypass Surgery in Moyamoya Disease**

Seiei Torazawa^1^, Satoru Miyawaki^1^, Hideaki Imai^1,2^, Hiroki Hongo^1^, Masahiro Shimizu^3^, Hideaki Ono^1^, Shotaro Ogawa^1^, Yu Sakai^1^, Satoshi Kiyofuji^1,4^, Satoshi Koizumi^1^, Daisuke Komura^5^, Hiroto Katoh^5^, Shumpei Ishikawa^5^, Nobuhito Saito^1^

^1^The University of Tokyo, Department of Neurosurgery, Faculty of Medicine, Tokyo, Japan

^2^Tokyo Shinjuku Medical Center, Department of Neurosurgery, Tokyo, Japan

^3^Kanto Neurosurgical Hospital, Department of Neurosurgery, Saitama, Japan

^4^Fuji Brain Institute and Hospital, Department of Neurosurgery, Shizuoka, Japan

^5^The University of Tokyo, Department of Preventive Medicine, Graduate School of Medicine, Tokyo, Japan

**Corresponding author:** **Satoru Miyawaki, MD, PhD**

E-mail: smiya-nsu@m.u-tokyo.ac.jp

**Online Resource 4** Basic characteristics of the enrolled patients (n=81)

| Sex (female) | 57 (70.4) |
| --- | --- |
| Age at initial operation |  |
| Median [IQR] (years) | 44 [37–52] |
| <16 years | 6 (7.4) |
| Hypertension | 29 (35.8) |
| Diabetes mellitus | 4 (4.9) |
| Dyslipidemia | 18 (22.2) |
| Smoking | 22 (27.2) |
| *RNF213* p.Arg4810Lys | 51 (63.0) |
| *RNF213* other variants | 20 (24.5) |

Data are presented as median [IQR] or n (%).

IQR, interquartile range.
