# Supplementary material for: Impact of genetic profiles on periventricular anastomosis following bypass surgery in moyamoya disease
Source: Neurosurg Rev. 2026 Apr 20;49(1):363. doi: 10.1007/s10143-026-04289-8 (PMC13092527; doi:10.1007/s10143-026-04289-8)
Supplement: Supplementary file 5 — Supplementary file5 (DOCX 19 KB) [file 10143_2026_4289_MOESM5_ESM.docx]

**Impact of Genetic Profiles on Periventricular Anastomosis Following Bypass Surgery in Moyamoya Disease**

Seiei Torazawa^1^, Satoru Miyawaki^1^, Hideaki Imai^1,2^, Hiroki Hongo^1^, Masahiro Shimizu^3^, Hideaki Ono^1^, Shotaro Ogawa^1^, Yu Sakai^1^, Satoshi Kiyofuji^1,4^, Satoshi Koizumi^1^, Daisuke Komura^5^, Hiroto Katoh^5^, Shumpei Ishikawa^5^, Nobuhito Saito^1^

^1^The University of Tokyo, Department of Neurosurgery, Faculty of Medicine, Tokyo, Japan

^2^Tokyo Shinjuku Medical Center, Department of Neurosurgery, Tokyo, Japan

^3^Kanto Neurosurgical Hospital, Department of Neurosurgery, Saitama, Japan

^4^Fuji Brain Institute and Hospital, Department of Neurosurgery, Shizuoka, Japan

^5^The University of Tokyo, Department of Preventive Medicine, Graduate School of Medicine, Tokyo, Japan

**Corresponding author:** **Satoru Miyawaki, MD, PhD**

E-mail: smiya-nsu@m.u-tokyo.ac.jp

**Online Resource 5** Basic information on *RNF213* rare variants and damaging variants detected in the GG with other variants group

| Position (GRCh38) | rsID | Amino acid change | AF in gnomAD | SIFT | PolyPhen-2 | MutationTaster | PROVEAN | CADD score |
| --- | --- | --- | --- | --- | --- | --- | --- | --- |
| 17:80345654 | rs761027115 | p.Gly2440Asp | 1.4×10^−5^ | Tolerated | Benign | Probably harmless | Deleterious | 22.5 |
| 17: 80345997 | rs138516230 | p.Asp2554Glu | 2.9×10^−5^ | Tolerated | Benign | Probably harmless | Neutral | 0.118 |
| 17:80346446 | rs146486225 | pArg2704Gln | 2.4×10^−5^ | Tolerated | Benign | Probably deleterious | Deleterious | 23.3 |
| 17:80347946 | rs2078368416 | p.His3204Arg | NA | Tolerated | Benign | Probably harmless | Neutral | 0.679 |
| 17:80358422 | rs375097553 | p.Met3666Thr | 3.4×10^−4^ | Deleterious | Probably damaging | Probably deleterious | Deleterious | 25.5 |
| 17:80372731 | rs138029774 | p.Pro4250Thr | 7.2×10^−3^ | Tolerated | Benign | Probably harmless | Neutral | 0.995 |
| 17:80376310 | rs148731719 | p.Ala4399Thr | 1.0×10^−2^ | Tolerated | Possibly damaging | Probably harmless | Neutral | 13.55 |
| 17: 80386819 | rs371441113 | p.Glu4950Asp | 6.4×10^−5^ | Tolerated | Probably damaging | Probably harmless | Neutral | 14.26 |

GG, wild-type of p.Arg4810Lys; rsID, reference SNP cluster ID; AF, allele frequency; gnomAD, Genome Aggregation Database; SIFT, Sorting Intolerant from Tolerant; PROVEAN, Protein Variation Effect Analyzer; CADD, combined annotation-dependent depletion (GRCh38-v1.6); NA, not applicable.
