# Supplementary material for: Impact of genetic profiles on periventricular anastomosis following bypass surgery in moyamoya disease
Source: Neurosurg Rev. 2026 Apr 20;49(1):363. doi: 10.1007/s10143-026-04289-8 (PMC13092527; doi:10.1007/s10143-026-04289-8)
Supplement: Supplementary file 6 — Supplementary file6 (DOCX 17 KB) [file 10143_2026_4289_MOESM6_ESM.docx]

**Impact of Genetic Profiles on Periventricular Anastomosis Following Bypass Surgery in Moyamoya Disease**

Seiei Torazawa^1^, Satoru Miyawaki^1^, Hideaki Imai^1,2^, Hiroki Hongo^1^, Masahiro Shimizu^3^, Hideaki Ono^1^, Shotaro Ogawa^1^, Yu Sakai^1^, Satoshi Kiyofuji^1,4^, Satoshi Koizumi^1^, Daisuke Komura^5^, Hiroto Katoh^5^, Shumpei Ishikawa^5^, Nobuhito Saito^1^

^1^The University of Tokyo, Department of Neurosurgery, Faculty of Medicine, Tokyo, Japan

^2^Tokyo Shinjuku Medical Center, Department of Neurosurgery, Tokyo, Japan

^3^Kanto Neurosurgical Hospital, Department of Neurosurgery, Saitama, Japan

^4^Fuji Brain Institute and Hospital, Department of Neurosurgery, Shizuoka, Japan

^5^The University of Tokyo, Department of Preventive Medicine, Graduate School of Medicine, Tokyo, Japan

**Corresponding author:** **Satoru Miyawaki, MD, PhD**

E-mail: smiya-nsu@m.u-tokyo.ac.jp

**Online Resource 6** Factors other than *RNF213* genotype associated with the score decrease of choroidal periventricular anastomosis in groups 1 and 2 (hemisphere-level analysis using mixed-effects models)

|  | P value |
| --- | --- |
|  | Choroidal PA (Gr1,2: n=52) |
| Age at surgery (≥16 vs. <16 years) | **0.003** |
| Donor development grade (fair/good/excellent) | 0.94 |
| Suzuki stage (stages 2, 3, and 4) | 0.14 |
| Sex | 0.68 |
| Hypertension | 0.48 |
| Diabetes mellitus | 0.71 |
| Hyperlipidemia | 0.49 |
| Smoking | 0.85 |
